# Cognitive Performance in Parkinson’s Spectrum Disorders: a comparative study of patients with Parkinson’s Disease, Parkinson’s Disease Dementia, Dementia With Lewy Bodies, Progressive Supranuclear Palsy, and Corticobasal Syndrome

**DOI:** 10.1177/10731911251339362

**Published:** 2025-05-24

**Authors:** Eva A. van Breugel, Esther van den Berg, Sanne Franzen, Judy van Hemmen, Harro Seelaar, Caroline van Heugten, Lize C. Jiskoot

**Affiliations:** 1 Erasmus MC University Medical Center, Rotterdam, The Netherlands; 2 Maastricht University, Maastricht, The Netherlands

**Keywords:** Parkinson’s spectrum, Parkinson’s disease, dementia with Lewy bodies, progressive supranuclear palsy, corticobasal syndrome, neuropsychological assessment

## Abstract

The Parkinson’s spectrum encompasses Parkinson’s disease (PD), PD dementia (PDD), dementia with Lewy bodies (DLB), progressive supranuclear palsy (PSP), and corticobasal syndrome (CBS). Clinical diagnosis mainly relies on progression over time and neuroimaging, biomarkers, and neurological observations, aided by neuropsychological assessment. Neuropsychological profiles and differences within the Parkinson’s spectrum have been understudied. This retrospective study analyzed mean performance and proportions of impairment of neuropsychological assessments of 212 patients in the Parkinson’s spectrum at an academic outpatient memory clinic. Patients with PD scored significantly higher than the other patient groups on most tests. The other patient groups showed limited significant differences from one another. The letter fluency test was most effective in distinguishing PD from the other disorders. The auditory verbal learning test was suitable to distinguish PDD and DLB from the other disorders. Results indicate considerable overlap in cognitive profiles across Parkinson’s spectrum disorders, suggesting neuropsychological assessment is valuable for assessing cognitive function, guiding post-diagnostic support, and monitoring progression, rather than differential diagnosis.

## Introduction

Parkinson’s Spectrum Disorders constitute a group of neurodegenerative disorders that share central motor symptoms such as bradykinesia, rigidity, tremor, and gait disturbances (Gerstenecker et al., 2013). In addition to Parkinson’s Disease (PD) and Parkinson’s Disease Dementia (PDD), the spectrum includes dementia with Lewy bodies (DLB), progressive supranuclear palsy (PSP), corticobasal syndrome (CBS), and multiple system atrophy (MSA). Notably, these latter syndromes exhibit a more rapid deterioration and a reduced response to levodopa medication than PD, underscoring the significance of early and accurate diagnosis for effective clinical management, prognosis monitoring, and patient perspective (Brown et al., 2010; Gomperts, 2016; Jabbari et al., 2020; Kessels et al., 2022). The complex, heterogeneous, and overlapping nature of Parkinson’s spectrum disorders poses a considerable challenge in the clinical distinction from one another. The characteristics of the Parkinson’s spectrum disorders are described below and summarized in Table 1.

**Table 1:** Characteristics of Parkinson’s Spectrum Disorders.

	PD	PDD	DLB	PSP	CBS	MSA
Motor symptoms	Bradykinesia, rigidity, rest tremor, postural instability	Bradykinesia, rigidity, rest tremor, postural instability	Bradykinesia, rigidity	Akinetic-rigid syndrome, ocular motor dysfunction, postural instability, early falls	Asymmetrical Parkinsonism, dystonia, myoclonus	Parkinsonism (sometimes asymmetrical), cerebellar ataxia abnormal posture
Cognition	EF, attention, mental processing speed, visuospatial functions	EF, attention, mental processing speed, visuospatial functions	EF, attention, mental processing speed, visuospatial functions, fluctuating cognition	EF, attention, mental processing speed, memory, language	EF, attention, fluency, memory visuospatial/-constructive functions	EF, attention, working memory, memory, visuospatial functions
Pathology	α-synucleinopathy	α-synucleinopathy	α-synucleinopathy	Tauopathy	Tauopathy	α-synucleinopathy
Other	No dementia	Dementia in the context of diagnosed PD	Dementia before/within one year of parkinsonism, hallucination, REM sleep behavioral disorder	Dementia	Asymmetric apraxia, cortical sensation loss, behavioral changes	Autonomic failure, orthostatic hypotension, respiratory disturbances, dementia not consistently found

*Note*. PD = Parkinson’s disease; PDD = Parkinson’s disease dementia; DLB = Dementia with Lewy Bodies; PSP = progressive supranuclear palsy; CBS = corticobasal syndrome; MSA = multiple system atrophy; EF = executive functions.

PD constitutes a clinical syndrome, encompassing motor, cognitive, and neuropsychiatric symptoms. The cognitive domains affected in PD commonly include executive function (EF), attention, mental processing speed, and visuospatial functioning (Fields, 2017; Watson & Leverenz, 2010). PDD is diagnosed when dementia, cognitive fluctuations, and hallucinations develop in the context of PD (Gomperts, 2016; Jellinger & Korczyn, 2018; Walker et al., 2015). PDD and DLB overlap substantially in both pathophysiology and clinical features and are therefore differentiated primarily by the timing of the onset of dementia relative to the development of symptoms of parkinsonism. In DLB, dementia develops before or within 1 year of parkinsonism (Gomperts, 2016; Walker et al., 2015). Cognitive domains that are affected in DLB include attention, executive function and visuospatial and constructive functions (Walker et al., 2015). Additionally, patients often present with fluctuating cognition, visual hallucinations, and parkinsonism (Walker et al., 2015). PSP is characterized by an akinetic-rigid syndrome, with oculomotor dysfunction, postural instability, falls early in the disease, and dementia (Burrell et al., 2014; Pilotto et al., 2017). Cognitive deficits in PSP include deficits in attention and mental processing speed, EF, memory retrieval, and language (Burrell et al., 2014; Peterson et al., 2021; Troster & Woods, 2003). CBS is recognized by asymmetric apraxia and/or cortical sensation loss, including an alien-hand sign (Gomperts, 2016). Patients with CBS can experience cognitive impairments in the domains of EF, attention, verbal fluency, visuospatial and/or -constructive abilities, and memory retrieval (Constantinides et al., 2019; Troster & Woods, 2003). Patients with CBS can also experience a “true” breakdown of language abilities rather than language problems due to impairments in EF and initiation (Peterson et al., 2021). Additionally, with CBS can experience changes in behavior and personality similar to those seen in frontotemporal dementia (FTD) (Burrell et al., 2014). Lastly, MSA is a rare disorder characterized by autonomic failure, Parkinsonism, and/or cerebellar ataxia (Fanciulli & Wenning, 2015). Cognitive deficits can also occur, particularly in executive functioning, but in some cases also in attention, (working) memory, and visuospatial functioning (Stankovic et al., 2014).

There are several differences between disorders in the Parkinson’s spectrum described in literature. PDD differs from PD in the severity of cognitive decline, including more impairments in memory and language understanding and production over time (Emre et al., 2007; Kehagia et al., 2010; Noe et al., 2004; Pagonabarraga & Kulisevsky, 2012). PDD and DLB have similar cognitive profiles, but patients with DLB often have more visuospatial impairments than patients with PDD (Oda et al., 2009; Walker et al., 2015; Yousaf et al., 2019). PSP is described to show the most significant executive dysfunction compared with other disorders in the Parkinson’s spectrum (Burrell et al., 2014). Relative to PD and MSA, cognitive problems in PSP emerge early in the disease course, are commonly more severe, and progress more rapidly (Fiorenzato et al., 2019; Troster & Woods, 2003). Furthermore, CBS overlaps most with PSP, but is distinguished by the presence of asymmetric apraxia and visuospatial problems (Burrell et al., 2014; Gomperts, 2016). Despite the documented distinctions between Parkinson’s spectrum disorders, research on the cognitive profiles of and differences within the Parkinson’s spectrum remains limited. Subtle cognitive differences between the conditions are often missed (Kessels et al., 2022). Currently, the clinical, neuropsychological distinction between syndromes mainly relies on disease progression (i.e., cognitive deterioration) over time, which is often difficult to accurately determine in clinical practice (Peterson et al., 2021). Additionally, relying on the progression of cognitive symptoms over time rather than at first presentation in the memory clinic makes the diagnostic process longer and more uncertain for patients and caregivers. For these reasons, diagnosis by the neurologist and/or geriatrician is accompanied by neuropsychological assessment, neuroimaging, clinical observations by the physician (neurologist and/or geriatrician) and (clinical) neuropsychologist, and sometimes also fluid biomarkers to rule out other neurodegenerative disorders, such as FTD or Alzheimer’s disease (Armstrong et al., 2013; Höglinger et al., 2017; McKeith et al., 2017; Postuma et al., 2015).

Thus, although research has been conducted on the cognitive symptoms of Parkinson’s spectrum disorders, there is limited literature comparing all syndromes (i.e., PD, PDD, DLB, PSP, CBS, and MSA) directly in one study. Research into the differences in cognitive profiles of these disorders could aid differential diagnosis. To address these clinical needs, the current study aimed to investigate the differences between cognitive profiles of Parkinson’s spectrum disorders. This study is primarily designed to aid neuropsychologists working in (academic) outpatient memory clinics. Therefore, rather than examining general cognitive profiles, this study analyzed the differences between Parkinson’s spectrum disorders on specific neuropsychological tests covering the major cognitive domains commonly used in (academic) outpatient memory clinics. The study aimed to investigate: (a) the cognitive profile of each disorder, (b) the differences on mean test performance and proportions of impairment, and (c) which neuropsychological tests can best distinguish between Parkinson’s spectrum disorders.

## Methods

### Participants

**Figure 1.**
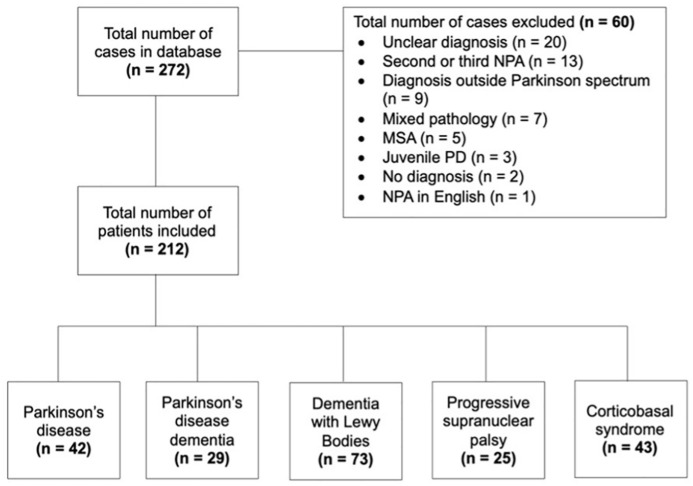
Flowchart of patient inclusion and exclusion. *Note*. NPA = neuropsychological assessment; MSA = multiple system atrophy; PD = Parkinson’s disease; MCI = mild cognitive impairment.

This retrospective, cross-sectional study included Dutch patients with a Parkinson’s spectrum disorder, assessed in the outpatient memory clinic of the Alzheimer Centre of the Erasmus MC University Medical Centre (Rotterdam, the Netherlands) between February 2013 and December 2023. Patients were included in the study if they (a) received a diagnosis of PD, PDD, DLB, PSP, or CBS, (b) had undergone neuropsychological assessment in Dutch as part of clinical care, and (c) gave written informed consent for the use of their clinical data for research purposes. The large majority of patients belonged to the White race. Patients were excluded from the study if Dutch was not their native language or if they had a mixed (e.g., PSP + CBS), uncertain, or no diagnosis. Importantly, as there were only five patients with MSA, this sample size was considered too small to be included in this study. Figure 1 depicts patient inclusion and exclusion. Out of the 272 patients retrieved from the database, 212 patients were included in the study. Sixty patients were excluded from the study, due to no or an uncertain dementia diagnosis, a dementia diagnosis other than a Parkinson’s spectrum disorder, an MSA diagnosis, a second or third neuropsychological assessment, mixed Parkinson’s spectrum diagnosis (e.g., PSP + CBS), a juvenile form of PD, or a neuropsychological assessment conducted another language than Dutch, to ascertain homogeneity in the patient groups. A sensitivity power analysis was conducted using G*Power (version 3.1.9.7; Faul et al., 2009) to determine the minimum detectable effect size for the planned analysis. The sensitivity power analysis was based on a fixed-effects, omnibus, one-way ANCOVA with five groups and three covariates. The analysis (total sample *n* = 212, α = .05, power = 0.80) indicated that the study was adequately powered to detect a minimum effect size of *f* = 0.21 (Cohen, 1988), which corresponds to a “moderate” effect.

### Procedure

Patients were seen out the outpatient memory clinic of the Alzheimer Centre of Erasmus MC University Medical Centre and movement disorder expertise centre. All participants underwent a standardized clinical interview and neurological or geriatric and neuropsychological assessment, in which the major cognitive domains were tested. Most participants underwent laboratory testing (lumbar puncture and/or blood sampling) and structural magnetic resonance imaging of the brain. These assessments were part of routine clinical care, meaning that the assessors were not blinded. Clinical diagnoses were made in weekly multidisciplinary meetings of the Department of Neurology and Alzheimer Centre of the Erasmus MC University Medical Centre, including for example, neurologists, geriatricians, neuropsychologists, radiologists, and psychiatrists. Clinical diagnoses and clinical (Mini-Mental State Examination (MMSE) score, Montreal Cognitive Assessment (MoCA) score, and disease duration) and demographic data (age, sex, and education) were retrieved from the electronic patient records. We report how we determined our sample size, all data exclusions, all manipulations, and all measures in the study.

### Ethics

All participants were part of a local biobank study, for which the Erasmus MC University Medical Centre ethics committee gave approval (MEC-2016-069). Patients gave written informed consent for study participation.

### Neuropsychological Assessment

MMSE (Folstein et al., 1975) and MoCA (Nasreddine et al., 2005) total scores were used as measures of global cognitive performance. Patients underwent an extensive, standardized neuropsychological assessment covering the major cognitive domains, including memory, attention and mental processing speed, EF, language, visuospatial, and -constructive abilities, and praxis. The tests included in this study are the following: the Auditory Verbal Learning Test (AVLT) (Saan & Deelman, 1986); Trail Making Test (TMT; part A and B, ratio-score B:A) (Schmand et al., 2003); Stroop color-word test (cards 1, 2, and 3, ratio score 3:2) (Schmand et al., 2003); semantic (animals) and letter fluency tests (D–A–T, K–O–M, or P–G–R—the Dutch equivalents of F–A–S) (Audenaert et al., 2000); the 60-item Boston Naming Task (BNT) (Heesbeen & van Loon-Vervoorn, 2001); Clock Drawing Test (Nishiwaki et al., 2004; Royall et al., 1998), and praxis test (van Heugten et al., 1999). An overview of the tests used and descriptions per test can be found in Appendix 1.

### Statistical Analysis

Following the procedure described in Fasnacht et al. (2023), MoCA scores were converted to MMSE scores in order to use one consistent measure of global cognitive performance in this study. MoCA to MMSE (and not the reverse) was chosen because the MMSE was administered to the majority of patients in this study (78%) and this transformation is considered the most accurate (Fasnacht et al., 2023). Analyses on demographic data were performed to determine differences between patient groups in age, sex, education level (according to Verhage, a Dutch educational scoring system, categorized into levels from 1 = *less than 6 years of primary education* to 7 = *academic education* [Duits, 2014]), MMSE total score, and disease duration (defined as the amount of years symptoms were present) at time of the neuropsychological assessment. An analysis of variance was used to compare age between groups. Kruskal–Wallis *H* tests were used to compare education level, MMSE total score, and disease duration, as these variables were not normally distributed. A chi-square test of independence was used to analyze differences in sex distribution. To ease interpretation, raw neuropsychological assessment test scores were transformed into standardized *Z* scores, corrected for age, sex, and education level, using test-specific normative data. The mean standardized scores were compared between patient groups using analyses of covariance (ANCOVA), with disease duration as covariate. Non-normally distributed data underwent logarithmic transformations to fit assumptions for the ANCOVA. Additionally, Chi-square tests of independence or Fisher–Freeman–Halton Exact tests (the latter in case of small expected counts) were used to compare proportions of exceptionally low, below average, and unimpaired (both low average and average) performance between patient groups. Performance in this context is defined according to conventions described in Hendriks et al. (2020), the Dutch adaptation of Guilmette et al. (2020) (Table 2) . All analyses were carried out using SPSS software (version 24.0, IBM Corp., Armonk, NY, USA). Corrections for multiple testing were applied by means of Bonferroni correction.

**Table 2:** Description of Standardized Cognitive Performance.

*Z*-score	Description
Lower or equal to −2	Exceptionally low
Between −2 and −1.4	Below average
Between −1.3 and −0.7	Low average
Higher or equal to −0.6	Average^a^

a For the purposes of this study, scores higher than average are not defined according Hendriks et al. (2020) but solely defined as average.

## Results

### Patient Characteristics

Demographic and clinical data of the final sample is shown in Table 3. There were more female patients in the CBS group (χ^2^[4] = 10.534, *p* = .032) than the other patient groups. Patients with PD were younger than patients with PDD, DLB, and CBS (*F*[4, 207] = 5.413, *p* < .001) and had higher MMSE total scores than patients with DLB and PSP (*H*[4] = 20.362, *p* < .001). Patients with PDD had higher education levels than patients with PSP (*H*[4] = 9.596, *p* = .048). Patients with PD and PDD had a longer disease duration at time of neuropsychological assessment than patients with DLB, PSP, and CBS (*H*[4] = 53.960, *p* < .001).

**Table 3:** Demographic Data. Values Indicate Mean ± Standard Deviation (Range) or Percentage.

	PD	PDD	DLB	PSP	CBS	Test Statistic	*p*-value
*n*	42	29	73	25	43		
Age (years)	63.4 ± 9.3 (40–79)	70.1 ± 5.9 (59–83)	69.9 ± 7.8 (50–89)	68.2 ± 5.2 (56–78)	68.9 ± 8 (47–85)	*F*[4, 207] = 5.413	<.001*
Sex (male)	30 (71.4)	21 (72.4)	54 (74)	17 (68)	20 (46.5)	χ^2^[4] = 10.53	.032*
Education (Verhage^a^)	5.1 (1.3)	5.4 (1.4)	4.9 (1.5)	4.5 (1.2)	4.7 (1.5)	*H*[4] = 9.596	.048*
MMSE (/30)	27.1 ± 2.9 (19–30)	25.8 ± 2.6 (21–30)	23.4 ± 4.0 (13–29)	24.0 ± 3.2 (15–28)	25.2 ± 3.9 (15–30)	*H*[4] = 20.362	<.001*
Disease duration (years)	9.8 ± 7 (0.5–35)	10 ± 6.6 (2–27)	4.5 ± 4.5 (0–27)	3.4 ± 1.8 (1–9)	3.4 ± 2.9 (0.5–13)	*H*[4] = 53.960	<.001*

*Note*. PD = Parkinson’s disease; PDD = Parkinson’s disease dementia; DLB = Dementia with Lewy Bodies; PSP = progressive supranuclear palsy; CBS = corticobasal syndrome; SD = standard deviation; MMSE = Mini-Mental State Examination.

a Dutch education system categorized into levels from 1 = *less than 6 years of primary education* to 7 = *academic schooling* (Duits, 2014).

* Significant *p*-values are denoted with an asterisk.

### Cognitive Performance Per Domain

#### Memory

On AVLT Encoding, ANCOVA showed that there were significant differences between the patient groups (*F*[4, 175] = 10.066, *p* < .001). Post-hoc tests with Bonferroni correction for multiple comparisons showed that patients with PD performed better than patients with PDD (*p* < .001) and DLB (*p* < .001). Additionally, patients with CBS performed better than patients with PDD (*p* = .001) and DLB (*p* < .001). The other groups did not differ significantly (*p* > .05). On AVLT Recall, ANCOVA showed that there were significant differences between the patient groups (*F*[4, 175] = 7.540, *p* < .001). Post-hoc tests showed that that patients with PD performed better than patients with PDD (*p* = .003) and DLB (*p* < .001). Patients with CBS also performed better than patients with DLB (*p* = .038) (Table 4). The other groups did not differ significantly (*p* > .05).

**Table 4:** Mean Performance on Each Test (With Standard Deviations in Brackets). Descriptions of Mean Standardized *z*-scores Are Denoted With Colors: Green = Average, Yellow = Low Average, Orange = Below Average, Red = Exceptionally Low.

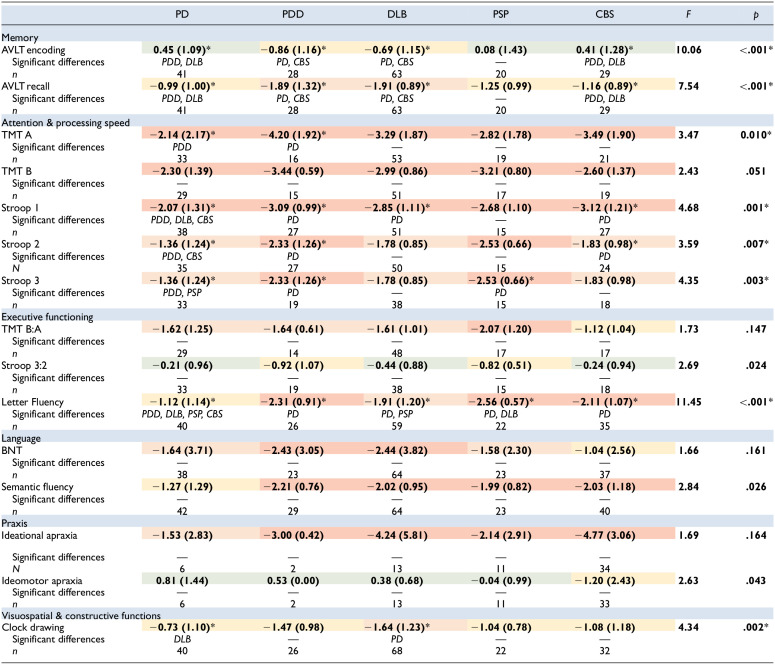

*Note*. PD = Parkinson’s disease; PDD = Parkinson’s disease dementia; DLB = Dementia with Lewy Bodies; PSP = progressive supranuclear palsy; CBS = corticobasal syndrome; AVLT = Auditory Verbal Learning Task; TMT = Trail-Making Test; BNT = Boston Naming Test.

* Significant *p*-values are denoted with an asterisk.

#### Attention and Processing Speed

On TMT A, ANCOVA showed that there were significant differences between the patient groups (*F*[4, 136] = 3.470, *p* = .010). Post-hoc tests showed that patients with PD performed better than patients with PDD (*p* = .007). The other groups were not significantly different (*p* > .05). On TMT B, ANCOVA showed no significant differences between the patient groups (*F*[4, 124] = 2.427, *p* = .051). On Stroop color-word card 1, ANCOVA showed significant differences between the patient groups (*F*[4, 152] = 4.684, *p* = .001). Post-hoc tests showed that patients with PD outperformed patients with PDD (*p* = .008), DLB (*p* = .018), and CBS (*p* = .004). There were no significant differences between the other patient groups (*p* > .05). On Stroop color-word card 2, ANCOVA showed significant differences between patient groups (*F*[4, 146] = 3.591, *p* = .007). Post-hoc tests showed that patients with PD performed better than patients with PDD (*p* = .031) and CBS (*p* = .037). No significant differences were found between the other patient groups (*p* > .05). On Stroop color-word card 3, ANCOVA also showed significant differences between patient groups (*F*[4, 117] = 4.354, *p* = .003). Post-hoc tests showed that patients with PD outperformed patients with PDD (*p* = .010) and PSP (*p* = .029). The other groups did not differ significantly (*p* > .05) (Table 4).

#### Executive Functioning

On the TMT B:A ratio, ANCOVA showed no significant differences between the patient groups (*F* 4, 119] = 1.732, *p* = .147). On the Stroop color-word test 3:2 ratio ANCOVA showed significant differences between patient groups (*F*[4, 118] = 2.689, *p* = .024). However, post-hoc tests with revealed no significant differences between specific patient groups. On the letter fluency task, ANCOVA showed significant differences between the patient groups (*F*[4, 177] = 11.453, *p* < .001). Patients with PD performed better than patients with PDD (*p* < .001), DLB (*p* = .012), PSP (*p* < .001), and CBS (*p* = .002) on post-hoc tests. Moreover, patients with DLB outperformed patients with PSP (*p* = .012). The other groups did not differ significantly (*p* > .05) (Table 4).

#### Language

ANCOVA showed no significant differences between the patient groups on the BNT-60 (*F*[4, 178] = 1.661, *p* = .161). On semantic fluency, ANCOVA showed significant differences between patient groups (*F*[4, 192] = 2.837, *p* = .026). However, after correcting for multiple comparisons, no statistically significant differences remained between specific patient groups (Table 4).

#### Praxis

No significant differences between patient groups on ideational apraxia in ANCOVA (*F*[4, 59] = 1.693, *p* = .164). ANCOVA did show significant differences between the patient groups on ideomotor apraxia (*F*[4, 59] = 2.631, *p* = .043). However, after correcting for multiple comparisons, no statistically significant differences between patient groups remained (Table 4).

#### Visuospatial and Constructive Functions

On the Clock Drawing test, ANCOVA showed significant differences between patient groups (*F* 4, 183] = 3.712, *p* = .006). Post-hoc analyses demonstrated that patients with PD performed better than patients with DLB (*p* = .021). There were no significant differences between the other patient groups (*p* > .05) (Table 4).

### Proportions of Cognitive Performance

#### Memory

**Figure 2.**
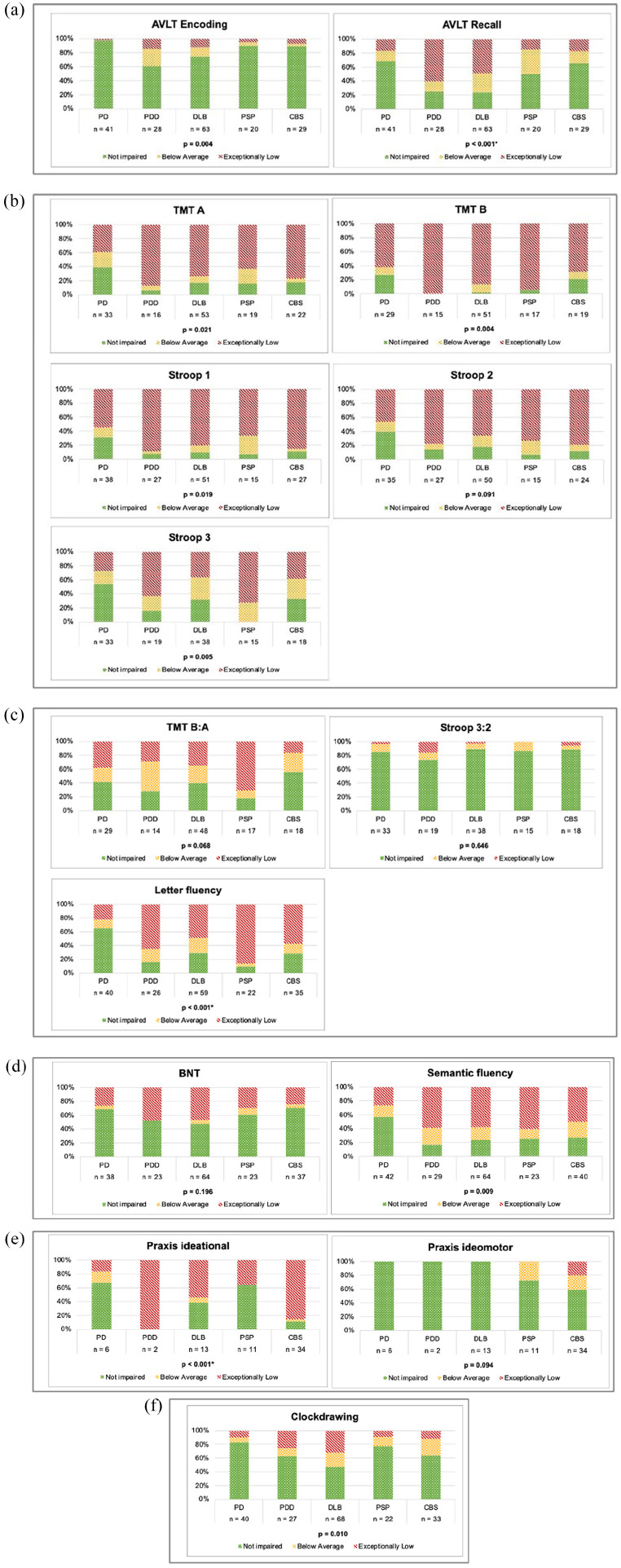
Proportions of performance. (a) Memory, (b) attention and processing speed, (c) executive functioning, (d) language, (e) praxis, and (f) visuospatial and -constructive functions. *Note*. PD= Parkinson’s disease; PDD= Parkinson’s disease dementia; DLB = Dementia with Lewy Bodies; PSP = progressive supranuclear palsy; CBS = corticobasal syndrome; AVLT = Auditory Verbal Learning Test; TMT= Trail making Test; BNT= Boston Naming Test.

There were significant differences in proportions of exceptionally low, below average, and unimpaired performance between the patient groups on AVLT Encoding (*p* = .004) (Figure 2a). However, when correcting for multiple comparisons, this difference was no longer significant (α = .003). Significant differences were found on the AVLT recall trial (χ^2^[8] = 39.05, *p* < .001).

#### Attention and Processing Speed

There were significant differences in proportional performance between patient groups on the TMT A (*p* = .021), TMT B (*p* = .004), Stroop color-word card 1 (*p* = .019), and Stroop color-word card 3 (*p* = .005) (Figure 2b). However, these differences were no longer significant after Bonferroni correction (α = .003). No significant differences between patient groups were found for Stroop color-word card 2 (*p* = .091).

#### Executive Functioning

No significant differences in proportional performance on the TMT B:A ratio (χ^2^[8] = 14.481, *p* = .068) or Stroop color-word test card 3:2 ratio (Fisher–Freeman–Halton Exact Test, *p* = .646) were found between patient groups. On letter fluency, PD patients showed less impairment than all other patient groups, also after correction for multiple testing (χ^2^[8] = 36.156, *p* < .001, Bonferroni, α = .003 (Figure 2c).

#### Language

No significant differences were found between the patient groups on proportional performance of the BNT-60 (Fisher–Freeman–Halton Exact test *p* = .196; Figure 2d). Significant differences were found in proportional performance on the semantic fluency test (χ^2^[8] = 20.339, *p* = .009), but this difference was no longer significant after correction for multiple comparisons (Bonferroni, α = .003).

#### Praxis

Patients with CBS showed more impairment than patients with PD and PSP on the ideational praxis test (Fisher–Freeman–Halton Exact Test, *p* < .001, corrected α = .003), but not on the ideomotor praxis test (Fisher–Freeman–Halton Exact Test, *p* = .094 (Figure 2e).

#### Visuospatial and Constructive Functions

A significant difference between the patient groups on the Clock Drawing test proportional performance was found (χ^2^[8] = 20.051, *p* = .01), but this difference was no longer significant after correction for multiple comparisons (Bonferroni, α = .003) (Figure 2f).

## Discussion

This retrospective study, spanning over a decade, investigated differences between patients with Parkinson’s spectrum Disorders (i.e., PD, PDD, DLB, PSP, CBS) on neuropsychological tests commonly used in outpatient memory clinics. The findings revealed that patients with PD overall performed better on the neuropsychological tests than all other patient groups. The largest differences were observed between patients with PD and patients with PDD and DLB. These differences were found on tests for memory, attention and processing speed, language, and visuospatial and constructive function, respectively. The letter fluency test distinguished best between PD and the other patient groups. Between the other Parkinson’s spectrum patient groups, not many significant differences were found, highlighting their overlapping manifestations on neuropsychological tests.

The finding that patients with PD showed the best cognitive performance in comparison to patients with other Parkinson’s spectrum Disorders is consistent with the existing literature (Duits, 2015). However, patients with PD *did* exhibit deficits on certain tests compared to norm data: of all tests included in the study, patients with PD showed the lowest mean performance on tests of attention and processing speed (TMT A and Stroop color-word test), which corresponds to findings from previous studies (e.g., Arroyo et al., 2021; Fields, 2017). Deficits in attention and processing speed are described to occur early in the disease course of PD and are among the cognitive domains that decline the most over time compared to other cognitive domains (Muslimović et al., 2009). Although patients with PD show *mental* slowness that is thought to be distinct from *motor* slowness, it is important to consider that the latter could have influenced patients’ performance on the TMT and Stroop color-word test (Arroyo et al., 2021; Hsieh et al., 2008; Sawamoto et al., 2002; Vlagsma et al., 2016). In the current study, these two types of slowness could not be separated, but in clinical practice, this could be achieved by, for instance, using the Delis–Kaplan Executive Functioning System (D-KEFS) version of the TMT (Delis et al., 2001). In this test, a separate motor speed task is included, which can be used to correct for the effects of motor speed in the parts of the TMT measuring speed of information processing.

EF deficits, often described in PD (Koerts et al., 2009; Kudlicka et al., 2011), were not uniformly found in our study. While approximately 60% of patients showed impaired or below mean concept shifting abilities (TMT B:A ratio), only 15% of patients showed impaired or below average response inhibition performance (i.e., Stroop 3:2 ratio). This coincides with previous research in which concept shifting was found to be affected in patients with PD, whereas response inhibition was intact (Koerts et al., 2009; Kudlicka et al., 2011). As an explanation of this, Koerts et al. (2009) argued that the subthalamic nucleus shows increased excitatory activity in PD, which reduces activation of the thalamus and prefrontal cortex, but leaves inhibitory responses intact. Subthalamic nucleus deep-brain stimulation is hypothesized to cause a release of the brake of the subthalamic nucleus on the thalamus and prefrontal cortex. Consequently, patients are shown to have lower inhibition abilities after having undergone deep-brain stimulation surgery, which is not observed in patients that have not undergone this procedure.

Additionally, previous literature described that patients with PD often show lower performance on the Clock Drawing test due to deficits in EF and visuospatial abilities (Fields, 2017; Scarpina et al., 2020; Srivastava et al., 2022). However, in the present study, only 20% of patients showed below-average or impaired performance. This inconsistent finding may be due to the heterogeneity of visuoconstructive tests and even a variety in versions and scoring systems for the Clock Drawing tests used in the literature (Liebermann-Jordanidis et al., 2022). The different scoring systems of the Clock Drawing test are associated with different types of grey matter damage, possibly reflecting different functions (Matsuoka et al., 2011). Therefore, in Parkinson’s spectrum disorders, it may be more insightful to use a visuoconstructive task such as the Rey-Osterrieth Complex Figure Task that has yielded more uniform results in the literature (Liebermann-Jordanidis et al., 2022; Osterrieth, 1944; Rey, 1941). Another important benefit is that for this test a separate scoring system is available for the planning of the figure, which could provide more insight into the cognitive mechanism (either deficits in EF, visuospatial abilities, or a combination of the two) underlying lower visuoconstructive test performance.

Significant differences in cognitive performance were most apparent between patients with PD and patients with PDD. The latter group performed significantly lower on tests of attention and processing speed, consistent with literature showing that these functions decline most over time in PDD (Muslimović et al., 2009). Furthermore, patients with PDD showed deficits in additional cognitive functions compared to patients with PD. Firstly, more patients with PDD showed impaired or below average performance on AVLT recall (75%) than patients with PD (32%). This deficit in free recall of recently learned information is also described in previous studies and seems to be specific to PDD, distinguishing it from PD (Emre et al., 2007; Kehagia et al., 2010). Secondly, patients with PDD showed more impaired or below average performance on the letter fluency task (85%) than patients with PD (35%), which could be a reflection of more impaired EF performance. Thirdly, compared to norm data, patients with PDD also showed an impairment in language as measured by the BNT and semantic fluency task, which was not found in patients with PD. This coincides with previous studies describing that language disorders arise in the transition from PD to PDD (Kehagia et al., 2010; Noe et al., 2004; Pagonabarraga & Kulisevsky, 2012). It is important to note that performance on these language tests did not significantly differ between patients with PD and PDD in the current study. The differences on the semantic fluency task were no longer statistically significant after correcting for multiple testing, and the lack of significant differences on the BNT may be due to the large standard deviations (suggesting overlapping test performance in the patient groups) found for this test.

In clinical practice, DLB is mostly separated from PDD by the timing of symptom development. In PDD, dementia develops in the context of established PD and in DLB dementia symptoms develop before the onset of parkinsonism (Walker et al., 2015). In line with this, in our study, patients with PDD and DLB exhibited similar cognitive profiles with deficits in attention, mental processing speed, memory, and language. Nevertheless, patients with DLB differed less from PD in tests of attention and mental processing speed than patients with PDD. A possible explanation for this could be fluctuations in cognition, specifically attention, that occur more often in DLB in comparison to PDD (Goetz et al., 2008). This may have resulted in slightly improved performance on tests of attention and mental processing speed of patients with DLB compared to patients with PDD. Moreover, patients with DLB showed the lowest performance and most impairments on the Clock Drawing test, and their mean performance was significantly lower than that of patients with PD. This lower performance in visuospatial functions could be linked to occipital and posterior dysfunction described in DLB (Oda et al., 2009; Yousaf et al., 2019). EF deficits are also often described as part of the cognitive profile of DLB, but this was not found in de current study and no significant differences were found on TMT B:A and Stroop 3:2 ratio between patients with DLB and the other patient groups (Johns et al., 2009; Walker et al., 2015). Nevertheless, patients with DLB showed lower performance on the letter fluency task than patients with PD. However, patients with DLB outperformed patients with PSP, indicating that patients with PSP—in line with previous research—exhibit more executive dysfunction than patients with DLB (Raimo et al., 2023).

Patients with PSP exhibited deficits in attention and mental processing speed, and verbal fluency, aligning with previous research (Troster & Woods, 2003). A higher percentage of patients showed an impairment in letter fluency (83%) than in semantic fluency (61%) (Burrell et al., 2014; Lange et al., 2003). The mean semantic fluency performance was lower than the mean performance on the BNT, and more patients showed exceptionally low performance on the semantic fluency task than on the BNT. This is in line with the suggested breakdown of EF, which influences language abilities, rather than a “true” breakdown of language function (Peterson et al., 2021). Additionally, 83% of patients with PSP showed below average to exceptionally low performance on TMT B:A, measuring concept shifting, supporting previous studies showing difficulties in EF in these patients (Burrell et al., 2014). Patients with PSP did not show profound deficits in memory and visuospatial and constructive abilities, contrasting prior studies in PSP (Burrell et al., 2014). Memory deficits are also suggested by some studies in relation to executive dysfunction (Parthimos & Schulpis, 2020). Additionally, patients with PSP showed a small number of significant differences from the other patient groups. This may be explained by the clinical heterogeneity and the high amount of initial incorrect diagnoses in PSP (Horta-Barba et al., 2021; Williams & Lees, 2009), which could have influenced our patient group and—as a result—cognitive data as well.

The neuropsychological profile of CBS is described to overlap with PSP (Burrell et al., 2014). Accordingly, in the current study, patients with CBS also showed deficits in attention and mental processing speed, and verbal fluency. Additionally, patients with CBS showed the lowest performance on both apraxia tests, although most differences were not significantly different from norm data. This is in line with literature describing asymmetric apraxia as a common distinguishing feature in CBS and a component of the diagnostic criteria (Gomperts, 2016). Nevertheless, this is also argued to be a source of bias as CBS is more likely to be diagnosed when apraxia is present, as it is believed to be a core symptom, but could also have led to misdiagnosis (Zadikoff & Lang, 2005). In our study, patients with CBS were more impaired on a test for ideational apraxia than on a test for ideomotor apraxia, which contradicts previous literature describing most impairments to lie in ideomotor apraxia and less so in ideational apraxia. This discrepancy could result from the fact that ideational apraxia is understudied in patients with CBS (Huey et al., 2009; Zadikoff & Lang, 2005). Moreover, previous studies describe non-fluent language characteristics in CBS (Burrell et al., 2014). In our study, this can be found in impaired verbal fluency performance, often seen in patients with CBS (Constantinides et al., 2019; Oliveira et al., 2017; Stockbridge et al., 2021). Additionally, visuoconstructive problems are well established in patients with CBS (Burrell et al., 2014) but were not prominent in the current study. Moreover, executive dysfunction, described in the literature (Murray et al., 2007), was not found in the current study, except for low performance on the letter fluency task. The difference between our findings and previous studies may be explained by the heterogeneity of CBS with various pathologies underlying, such as PSP, AD, FTD, and DLB (Boeve, 2011; Zadikoff & Lang, 2005). Episodic memory seems to be relatively preserved in CBS, which was also found in the current study, underscored by significantly higher performance in patients with CBS than those with PDD and DLB (Murray et al., 2007).

The letter fluency test was most effective at distinguishing PD from other Parkinson’s spectrum disorders. This is consistent with previous studies highlighting that the verbal fluency test is sensitive in distinguishing patients with PSP from patients with PD (Burrell et al., 2014; Gupta et al., 2017; Lange et al., 2003; Rittman et al., 2013; Santangelo et al., 2018). The role of the letter fluency task in differentiating other Parkinson’s spectrum Disorders from PD has been less investigated. Additionally, AVLT encoding and recall distinguished patients with DLB and PDD from patients with PD and the other Parkinson’s spectrum Disorders. TMT B:A and Stroop 3:2 ratios, used to measure EF, were not useful in differentiating the different Parkinson’s spectrum disorders included in our study. Moreover, it is well established that executive dysfunction occurs in the Parkinson’s spectrum (Troster & Woods, 2003), but patients exhibited limited impairments on the Stroop 3:2, indicating possible limited clinical applicability of this test.

A few limitations must be considered when interpreting the results of this study. Firstly, the study’s retrospective and cross-sectional design involved analyzing patients referred to for neuropsychological assessment, commonly due to suspected cognitive impairment, which could have led to an overestimation of cognitive impairment. Therefore, it is possible that the patient groups presented in the current study do not represent the overall Parkinson’s spectrum patient population, but rather a patient selection referred for neuropsychological assessment only. Therefore, future studies should prospectively include all different types of Parkinson’s spectrum patients, rather than only those referred for neuropsychological assessment. Secondly, only native Dutch-speaking, mostly White patients were included in the study to prevent language and cultural influences on test performance (Harry & Crowe, 2014; Tan et al., 2021). This, however, limits the generalizability of findings to other cultural or ethnic populations. Patients who were not native Dutch-speakers underwent a different, cross-cultural test protocol (TULIPA study; Franzen et al., 2023). Future studies could research differences between disorders within the Parkinson’s spectrum using a cross-cultural neuropsychological test battery to increase generalizability. Additionally, we used Dutch norms to due to differences in culture on neuropsychological test performance. However, for the Clock Drawing test, no Dutch norms were publicly available, and therefore we used British norms. Although performance on the Clock Drawing test is culturally influenced, language does not have a substantial effect (Maestri et al., 2023); therefore, we consider the use of these norms acceptable. Thirdly, the praxis test specifically had small sample sizes compared to the other neuropsychological tests included in our study, as it is not part of the core battery of neuropsychological tests commonly used in our outpatient memory clinic and was only administered when apraxia was suspected (thus, most often in patients with suspected CBS). Consequently, performance on the praxis test is likely to be lower in this study than the general patient population. Fourthly, the relative differences in the sizes of the patient groups have reduced the power of the analyses, making it equivalent to that of the smallest group. Additionally, corrections for multiple comparisons may have introduced an additional decrease in power. Nevertheless, we regard our study as sufficiently powered to detect a minimum effect size of *f* = 0.21. Lastly, patients were referred to for neuropsychological assessment at different stages of their disease, which is reflected in the wide ranges in disease duration. In these neurodegenerative conditions, symptoms can vary as the disease progresses, which may have caused heterogeneity on test outcomes within patient groups. Disease duration was included as a covariate to account for this variability. However, we also acknowledge that disease duration in years does not reflect the same stage of disease for different disorders (e.g., Fiorenzato et al., 2019). Despite this limitation, no standardized method currently exists to adjust for disease stage across multiple disorders. Therefore, we opted for this approach as the most appropriate solution given the available data.

Based on the methods and results of the current study, future studies should consider similar examinations using different neuropsychological tests. Due to the retrospective nature of the study, we only included tests for which sufficient data were available. Future studies could consider analyses of different tests, such as different types of memory (stories or visual) or fine motor function. Moreover, norm data of the recognition trial of the AVLT were not freely available, which is why we chose not to include this test in the study. However, it would be interesting to examine both recall and recognition of the AVLT in the Parkinson’s spectrum in the future. Moreover, in order to prevent cultural influences on test performance (such as those in familiarity with items on the BNT; Harry & Crowe, 2014), the current study only included native Dutch-speaking patients. Patients who were not native Dutch underwent a different, cross-cultural protocol (Franzen et al., 2023). Future studies could research differences between disorders within the Parkinson’s spectrum using a cross-cultural neuropsychological test battery as well.

## Conclusion

This study indicates that the neuropsychological assessment does have the capacity to aid in differential diagnosis, specifically between PD and other related disorders in the Parkinson’s spectrum. Patients with PD namely exhibited significantly better performance on the neuropsychological tests than the other patient groups. Moreover, the letter fluency test showed most significant differences in the Parkinson’s spectrum. Additionally, the AVLT is useful in differentiating DLB and PDD from other disorders. Nevertheless, neuropsychological assessment potentially has limited ability in providing a differential diagnosis within the other disorders in the Parkinson’s spectrum, as not many statistically significant differences were found between PDD, PSP, DLB, and CBS. This suggests that the role of neuropsychological assessment within the Parkinson’s spectrum should be more focused on supporting psycho-education, treatment strategies and family guidance, rather than the differential diagnostic process itself (Troster & Woods, 2003). Because the current study was retrospective and mostly included patients referred for neuropsychological assessment who were suspected of having major cognitive disorders, future research can further uncover differences in the entire Parkinson’s spectrum population, irrespective of disease stage—rather than only those referred for neuropsychological assessment.

## Appendix

**Appendix 1:** Overview of Neuropsychological Tests.

Cognitive domain	Test	Subtest	Explanation	Norms used
Memory	AVLT	Encoding	A list of fifteen unrelated words is read out loud to patients in five trials. Patients are instructed to remember the list and name all the words they remember in each trial. The total score constitutes the total number of words remembered in the five trials together (max. 75).	Schmand et al. (2012)
		Recall	After a delay of fifteen to twenty minutes, participants are asked which words they remember from the list they learned earlier. The total number of words remembered constitutes the total score (max. 15).	
Attention and processing speed	TMT	A	Participants are instructed to connect numbers 1–25 depicted in circles as fast as possible. The total score is the time taken to complete the task (max. 300 s).	Schmand et al. (2012)
		B	Participants are instructed to connect numbers 1–13 as well as letters A–L depicted in circles while alternating numbers and letters, as fast as possible. The total score is the time taken to complete the task (max. 300 s).	
	Stroop color-word test	1	Participants are instructed to read consecutive four color names (red, blue, green, yellow) out loud as fast as possible. The total score is the time taken to complete the task (max. 300 s).	Schmand et al. (2012)
		2	Participants are instructed to name the color of consecutive blocks (red, blue, green, yellow) out loud as fast as possible. The total score is the time taken to complete the task (max. 300 s).	
		3	Written color names are printed in a non-corresponding color ink. Participants are instructed to name the color of the ink of consecutive words as fast as possible. The total score is the time taken to complete the task (max. 300 s).	
Executive functioning	TMT	B:A	A ratio score of TMT part B divided by part A is calculated to test concept-shifting ability.	Schmand et al. (2012)
	Stroop color-word test	3:2	A ratio score of Stroop card 3 divided by card 2 is calculated to test inhibition ability.	Schmand et al. (2012)
	Fluency	Letter	Participants are instructed to name as many words as they can that start with a specific letter for one minute (D–A–T; K–O–M; P–G–R, the Dutch equivalent of F–A–S). Total score is the sum of the three letters.	Schmand et al. (2012)
Language	BNT		Drawn images of everyday objects are presented. Participants are instructed to name the objects depicted in one word. The score is calculated by the total number of objects named correctly (max. 60).	Marien et al. (1998)
	Fluency	Semantic (animals)	Participants are instructed to name as many animals as possible in one minute. Total score is the total amount of animals named.	Schmand et al. (2012)
Praxis	Praxis test	Ideational	Ideational apraxia is the inability to carry out sequences of actions related to everyday object use. Participants are given several objects and asked how to show how one would use these objects.	van Heugten et al. (1999)
		Ideomotor	Ideomotor apraxia is the inability to perform imitations of gestures correctly. Participants are instructed to imitate movements performed by the neuropsychologist.	
Visuospatial/constructive abilities	Clock drawing Test		Participants are instructed to draw a clock, including its numbers and arms. Scoring principles according to Royall et al. (1998) are used (max. 14).	Royall et al. (1998)
